# LincROR Mediates the Suppressive Effects of Curcumin on Hepatocellular Carcinoma Through Inactivating Wnt/β-Catenin Signaling

**DOI:** 10.3389/fphar.2020.00847

**Published:** 2020-07-02

**Authors:** Jiang Shao, Chuan-Jian Shi, Yun Li, Feng-wei Zhang, Fei-fei Pan, Wei-ming Fu, Jin-fang Zhang

**Affiliations:** ^1^ Lingnan Medical Research Center, Guangzhou University of Chinese Medicine, Guangzhou, China; ^2^ Key Laboratory of Orthopaedics and Traumatology, The First Affiliated Hospital of Guangzhou University of Chinese Medicine, The First Clinical Medical College, Guangzhou University of Chinese Medicine, Guangzhou, China; ^3^ School of Pharmaceutical Sciences, Southern Medical University, Guangzhou, China; ^4^ Guangdong Provincial Key Laboratory of New Drug Screening, School of Pharmaceutical Sciences, Southern Medical University, Guangzhou, China; ^5^ Shenzhen Traditional Chinese Medicine Hospital, the Fourth Clinical Medical College of Guangzhou University of Chinese Medicine, Shenzhen, China

**Keywords:** curcumin, hepatocellular carcinoma, lincROR, apoptosis, Wnt/β-catenin signaling

## Abstract

As one of the leading causes of cancer-related death in the world, hepatocellular carcinoma (HCC) has continued to attract growing attention in recent decades. The use of traditional Chinese herbs in medicine has been practiced for thousands of years, and holds the potential of being a possible treatment for HCC. Curcumin, a bioactive ingredient derived from *Curcuma longa*, exhibits anti-tumor activity in various cancers. Although the effects of Curcumin on HCC have been elucidated, the underlying mechanism remains unclear. In the present study, Curcumin was demonstrated to inhibit the proliferation of HCC cells *via* inducing cell cycle arrest and apoptosis. Several previously reported lncRNAs related to tumorigenesis were chosen for examination of their expression profiles, and lincROR was found to be the most down-regulated in the Curcumin-treated HCC cells. Furthermore, Curcumin was found to decrease β-catenin expression and induce the inactivation of Wnt/β-catenin signaling. Therefore, Curcumin suppressed tumor growth through a lincROR/β-catenin regulatory pattern. In conclusion, our results demonstrated that Curcumin suppressed the cell proliferation *via* the down-regulation of lincROR and inactivation of Wnt/β-catenin signaling, suggesting that it may be a potential anti-cancer candidate for HCC patients with activated Wnt/β-catenin signaling.

## Introduction

As the most common type of liver cancer, hepatocellular carcinoma (HCC) is the third most frequent cause of cancer-related death in the world (Okuda, 2000; Jemal et al., 2005). Although the mortality rate in cancers has been decreasing recently, HCC is still one of the leading causes of death and remains a serious health problem in most countries (Bosch et al., 2004). Therefore, the development of more effective therapies is imperative and of high clinical significance for HCC patients. During recent years, the use of traditional Chinese medicine (TCM) has become increasingly popular in cancer therapeutics. Some medicines within TCM have been reported to serve as effective anti-cancer drugs or adjuvants to alleviate cancer progression and to improve the life quality of patients (Hu et al., 2013). Curcumin, a bioactive phenol, is isolated from *Curcuma longa,* which has been used for hundreds of years (Tsuda, 2018). Mounting evidence has revealed that Curcumin exhibited anti-cancer properties in various cancers, such as colorectal cancer, breast cancer, prostate cancer, HCC, etc. (Golonko et al., 2019; Vallée et al., 2019). However, the detailed underlying mechanisms of this anti-cancer effect remain obscure.

In recent years, non-coding RNAs, especially long non-coding RNAs (lncRNAs), have attracted more and more attention in research fields. They have been identified as important and powerful regulators in multiple diseases, including carcinogenesis (Mercer et al., 2009). Series of lncRNAs, such as H19, Hotair, and HOTTIP, have been demonstrated to serve as oncogenes to promote cancer development and metastasis (Gupta et al., 2010; Fan et al., 2018; Lecerf et al., 2019). The human lincROR is a recently identified lncRNA which negatively regulated the expression of p53 and inhibited cell cycle and apoptosis (Zhang et al., 2013), indicating its significant role in tumorigenesis.

In the present study, Curcumin was found to suppress tumorigenesis *via* inducing cell cycle arrest and apoptosis in HCC. Further investigation of underlying mechanisms demonstrated that lincROR was down-regulated and thus induced the inactivation of Wnt/β-catenin signaling. Therefore, our study provides a novel molecular mechanism of Curcumin in liver cancer progression and helps to generate a promising therapeutic candidate for cancer patients with aberrantly activated Wnt/β-catenin signaling.

## Material and Methods

### Preparation of Curcumin

Curcumin was purchased from sigma laboratory (Sigma-Aldrich, St. Louis, MO, USA) with purity over 99%.

### Cell Culture

Two HCC cell lines, SMMC7721 and Huh-7, and an immortalized non-tumorigenic cell line, LO2, were cultured in Dulbecco's modified Eagle's medium (DMEM, GIBCO, USA) supplemented with 10% fetal bovine serum (FBS, GIBCO) and 1% penicillin/streptomycin (P/S, GIBCO) in a humidified incubator at 37°C with 5% CO_2_. All the following experiments were repeated in triplicate, if no special indication is mentioned.

### Cell Proliferation

The SMMC7721 and Huh-7 cells were seeded in a 96-well plate and treated with various concentrations of Curcumin for 24, 48, and 72 hours. 10μl methylthiazoletetrazolium (MTT) solution (5mg/ml) was incubated for another four hours. Then the medium was removed, and 100μl dimethylsulfoxide (DMSO) was added to dissolve the formazan crystals. The absorbance was measured at 570nm with a Multiskan FC plate reader (Thermo Scientific, USA).

### Colony Formation Assays

Cells were seeded in a 6-well plate at 500 cells/well and incubated with 16μM Curcumin for two weeks. After being fixed with methanol, the colonies were stained with Giemsa for 20 min. ImmunoSpot analyzers (CTL, USA) were used to quantify the colonies.

### Apoptosis Assays

Cells were incubated with 16μM Curcumin for 48 hours and collected for flow cytometry examination. The apoptotic cells were quantified with an FITC-labeled Annexin V/propidium iodide (PI) Apoptosis Detection Kit (KeyGEN, China), as mentioned before (Fu et al., 2015).

### Cell Cycle Analysis

Cells were treated with 16μM Curcumin for 48 hours, and then were fixed in 70% ethanol. Then, the samples were stained with the dye from the Cell Cycle Detection Kit (KeyGEN), and the cells were subjected to flow cytometry examination (Beckman, Pasadena, CA).

### The lincROR Overexpressing Stable Cells

The SMMC-7721 cells with lincROR overexpression were generated using the retrovirus-mediated gene delivery system as previously described (Feng et al., 2018). Briefly, the retrovirus was generated in 293T cells by co-transfecting with pBabe-lincROR (plincROR) vector and the viral packaging vector pCL-Ampho. The pBabe vector (pVector) was used as the control. The SMMC-7721 cells were infected by retroviral particles, and the stable cells were developed by Puromycin (Sigma-Aldrich, USA) selection.

### Luciferase Activity Assays

HCC cells were seeded in 12-Well plates and co-transfected with a TOPflash luciferase reporter (1.25ug) by Lipofectamine 3000 (Invitrogen, USA). With 28-hour Curcumin treatment, luciferase activity was determined using the luciferase reporter assay system (Promega, USA) (Liang et al., 2019). Each experiment was repeated in triplicate.

### Quantitative Real Time PCR (qPCR)

Total RNA was extracted by Trizol reagent (Invitrogen, USA), and the cDNA was generated with PrimeScript RT Reagent Kit (TaKaRa, Japan). The qPCR analyses were conducted using a power up SYBR Green PCR master mix (Thermo Fisher Scientific) on a Light-Cycler480 System (Roche, Basel, Switzerland). The primers were listed in **Table 1**.

**Table 1:** Primers for qRT-PCR.

Gene	Forward Primers (5'-3')	Reverse primer(5'-3')
β-catenin	CCGTTCGCCTTCATTATGGA	GGCAAGGTTTCGAATCAATCC
CD44	TCAGAGGAGTAGGAGAGAGGAAAC	GAAAAGTCAAAGTAACAATAACAGTGG
c-Myc	TTCGGGTAGTGGAAAACCAG	CAGCAGCTCGAATTTCTTCC
GAPDH	GCACCACCAACTGCTTAGCA	TCTTCTGGGTGGCAGTGATG
HOTTIP	CCTAAAGCCACGCTTCTTTG	TGCAGGCTGGAGATCCTACT
HULC	TTCACCAGGCTGATAGTCCG	ACACGTCCTTCCCATAAACCC
H19	TGCTGCACTTTACAACCACTG	ATGGTGTCTTTGATGTTGGGC
Oct3/4	TCGAGAACCGAGTGAGAGGC	CACACTCGGACCACATCCTTC
LincROR	CTGGCTTTCTGGTTTGACG	CAGGAGGTTACTGGACTTGGAG

### Immunoblotting Analysis

The total proteins were extracted using RIPA solution. Nuclear protein fraction was extracted by the nuclear and cytoplasmic extraction kit (Invent, USA). The proteins were separated by 10% sodium dodecyl sulphate polyacrylamide gel electrophoresis (SDS-PAGE) and transferred to PVDF membranes (Millipore, Billerica, MA). After blocking, PVDF membranes were incubated with specific antibodies against β-catenin (1:2000; Cell Signaling Technology, USA), GADPH (1:2000; Cell Signaling Technology, USA), and Lamin A/C (1:1000; Cell Signaling Technology, USA) overnight, followed by the HRP-labelled corresponding IgG (Merck, Germany). Finally, the chemiluminescence (ECL, USA) was used to visualize the bands and the quantitative densitometries were determined with Image J.

### Immunofluorescence Analyses

After treatment with Curcumin for 72 hours, HCC cells were incubated with the anti-β-catenin antibody (1:100; Cell Signaling Technology, USA) overnight. They were then incubated with anti-rabbit IgG-Alexa Fluor 594 (Absin, Beijing, China) at 1:2000 dilution in the dark at 37°C for one hour. After the cell nuclei were labeled with DAPI (Beyotime, Shanghai, China), images were captured using a Zeiss Axiophot 2 microscope.

### Statistical Analysis

Data are presented as mean±SEM. Two-tailed unpaired Student's t test was employed to determine the statistical significance of differences. Differences were considered significant when P-values <0.05.

## Results

### Curcumin Significantly Suppressed Cell Viability in HCC Cells

**Figure 1:**
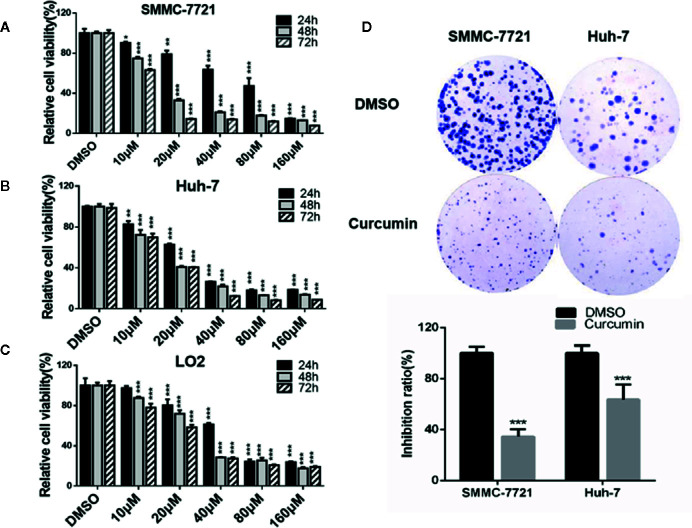
Curcumin inhibited cell proliferation in HCC cells. A-C, SMMC-7721, and Huh-7 cells were treated with serial concentrations of Curcumin, and the effects of Curcumin on cell proliferation were measured by MTT assays at 24, 48, and 72 hours **(A–C)**. **(D)** SMMC-7721 and Huh-7 cells were treated with Curcumin for about 14 days and the colony formation was examined. *P < 0.05, **P < 0.01, ***P < 0.001, compared with DMSO.

Curcumin has been reported to inhibit cell proliferation in multiple cancers (Golonko et al., 2019; Vallée et al., 2019). To validate this conclusion in our study, two HCC cells, SMMC-7721 and Huh-7, and one non-tumorigenic cells, LO2, were exposed to the concentrations of Curcumin varying from 0 to 160µM. As shown in **Figures 1A–C**, Curcumin significantly suppressed the two HCC cells' proliferation in dose-dependent manners. Compared with the effect of Curcumin on two HCC cells, it exhibited a weaker effect on cell viability in the LO2 cells (**Figure 1C**). The IC50 value of curcumin for 48 hours in SMMC-7721 cells was 15.41µM and it was 13.98µM in Huh-7 cells; we therefore selected 16µM for the further investigation. Moreover, the suppressive effect of Curcumin was further assessed by colony formation assays, and the results validated its anti-tumor function in HCC cells (**Figure 1D**).

### Curcumin Induced Cell Cycle Arrest and Apoptosis in HCC Cells

**Figure 2:**
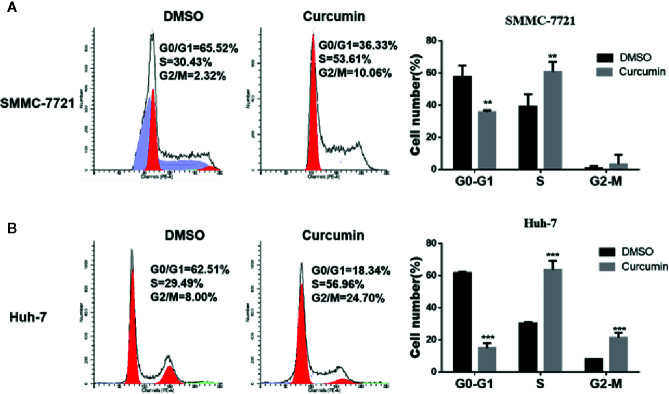
Curcumin induced cell cycle arrest in HCC cells. SMMC-7721 **(A)** and Huh-7 **(B)** cells were treated with 16μM Curcumin for 48 hours, then harvested and subjected to cell cycle analyses. **P < 0.01, ***P < 0.001, compared with DMSO.

**Figure 3:**
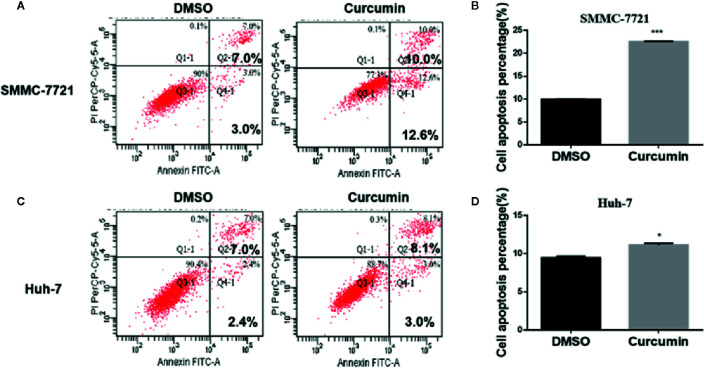
Curcumin induced apoptosis in HCC cells. SMMC-7721 **(A, B)** and Huh-7 **(C, D)** cells were incubated with 16μM Curcumin for 48 hours, and the apoptotic cells were examined by Annexin V-FITC and PI double staining. **(A, C)**, The results of one such assay; and **(B, D)**, mean ± SD of three independent experiments. *P < 0.05, ***P < 0.001, compared with DMSO.

In the subsequent experiments, the Curcumin-treated HCC cells were subjected to flow cytometry examination for cell cycle analysis. As shown in **Figure 2**, Curcumin induced an increased percentage of cells in the S phase and fewer cells in G0-G1 phase (**Figures 2A, B**), indicating that its anti-proliferation effect partially resulted from S-phase arrest. The quantitative apoptotic cells induced by Curcumin were also monitored. Compared with the DMSO-treated group, the Curcumin-treated group exhibited more apoptotic cells in SMMC-7721 cells (**Figures 3A, B**) and Huh-7 cells (**Figures 3C, D**). These results suggested that Curcumin suppressed cell proliferation partially *via* the induction of cell cycle arrest and apoptosis.

### Curcumin Suppressed the LincROR Expression in HCC Cells

**Figure 4:**
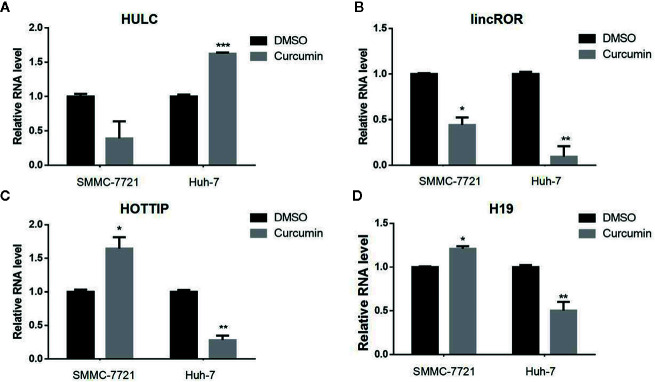
lincROR was the most changeable candidate in Curcumin-treated HCC cells. The two HCC cells were incubated with Curcumin for 48 hours, and several lncRNAs were chosen to examine their expression by qRT-PCR assays **(A–D)**. LincROR was the most dramatic change candidate in these treated cells. *P < 0.05; **P < 0.01; ***P < 0.001, compared with DMSO.

Many lncRNAs have been demonstrated to serve as critical regulators in tumorigenesis (Bhan et al., 2017). To identify the putative lncRNAs involved in this process, four previously reported lncRNAs including HULC (**Figure 4A**), lincROR (**Figure 4B**), HOTTIP (**Figure 4C**), and H19 (**Figure 4D**) were chosen and subjected for qRT-PCR examination to check their expression profiling. Among these candidate lncRNAs, only lincROR presented significant down-regulation in Curcimin-treated HCC cells (**Figure 4B**), and thus it was chosen for further investigation.

### Curcumin Blocked the Activation of Wnt/β-Catenin Signaling in HCC Cells

**Figure 5:**
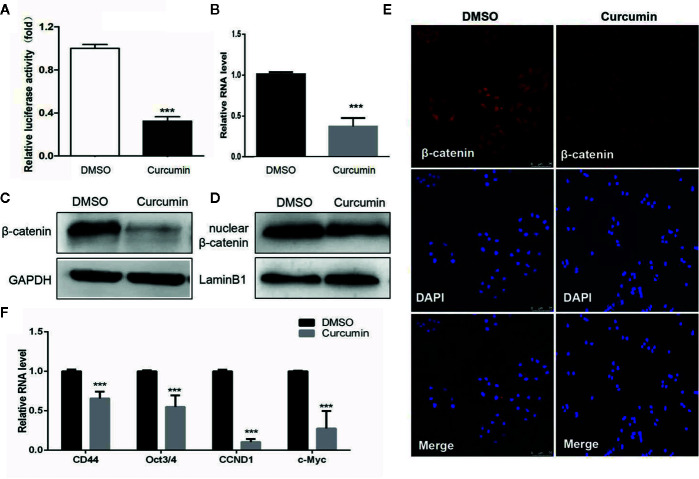
Curcumin induced the inactivation of the Wnt/β-catenin signaling. **(A–D)** With Curcumin treatment for 48 hours, the TOPflash luciferase activity was examined in SMMC-7721 cells **(A)**. The expression of β-catenin in SMMC-7721 cells was determined at mRNA level **(B)**. The expression of total β-catenin **(C)** and intranuclear β-catenin **(D)** were examined in SMMC-7721 cells at protein level. Lamin A/C (nuclear expression) and GADPH (cytoplasmic expression) were used as the loading controls. **(E)** β-catenin was detected in SMMC-7721 cells with 48 hours' treatment by immunofluorescence staining (100×). **(F)** The downstream targets of the Wnt/β-catenin pathway in Curcumin-treated SMMC-7721 cells were examined by qRT-PCR assays. ***P < 0.001, compared with DMSO.

The Wnt/β-catenin signaling is an important mechanism for cell proliferation, while its abnormal activation could lead to the development of various cancers (Anastas and Moon, 2013). To determine whether this signaling could be involved in this process, the Wnt signaling reporter TOPflash, which contains three binding sites for TCF and β-catenin, was transfected into the Curcumin-treated HCC cells. The results showed that Curcumin significantly suppressed the luciferase activity of TOPflash in SMMC-7721 cells (**Figure 5A**). Furthermore, the total β-catenin expression was reduced by Curcumin in SMMC-7721 cells at mRNA and protein levels (**Figures 5B, C**, **Supplementary Figure S1**). It is well known that β-catenin could be translocated from the cytoplasm to the nucleus, thus leading to the suppression on its transcription. We therefore examined the expression of the nuclear β-catenin and it was shown that less nuclear β-catenin was enriched in the Curcumin-treated HCC cells (**Figure 5D**, **Supplementary Figure S1**). Further immunofluorescence investigation also demonstrated that nuclear β-catenin was decreased by Curcumin in HCC cells (**Figure 5E**), and the quantitative analyses were showed in **Supplementary Figure S2**. Several downstream target genes of Wnt/β-catenin signaling, such as CD44, Oct3/4, CCND1, and c-Myc, were examined and the results showed that their expression was suppressed by Curcumin in SMMC-7721 cells (**Figure 5F**). All these results indicated that Curcumin suppressed the β-catenin expression, and thus induced the activation of the canonical Wnt/β-catenin signaling.

### Curcumin Suppressed Cell Viability Through LincROR/β-Catenin Regulatory Pattern

**Figure 6:**
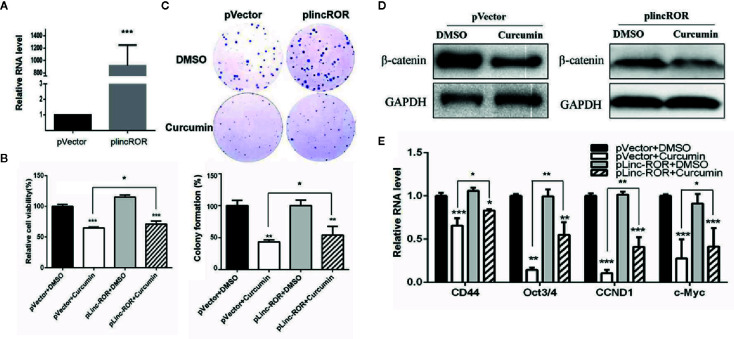
lincROR overexpression reversed Curcumin-induced growth inhibition and inactivated Wnt/β-catenin signaling. **(A)** The expression of lincROR in lincROR overexpressing SMMC-7721 cells was measured by qRT-PCR examination. ***P<0.001, compared with pVector. **(B, C)** The cell viabilities **(B)** and colony formation **(C)** was measured with Curcumin 48-hour treatment in lincROR overexpressing SMMC-7721 cells. **(D)** The protein level of β-catenin was examined by Western blotting after 48-hour treatment of Curcumin in lincROR overexpressing SMMC-7721 cells. **(C)** Other Wnt/β-catenin downstream target genes were examined by qRT-PCR with the same treatment in lincROR overexpressing SMMC-7721 cells. *P < 0.05; **P < 0.01; ***P < 0.001; compared with pVector+DMSO.

In our previous studies, lincROR has been demonstrated to activate Wnt/β-catenin signaling so as to promote osteogenesis in human mesenchymal stem cells (Feng et al., 2018). In this study, we firstly silenced lincROR by using the specific shRNA plasmid to test whether the reduced expression could mimic the suppressive effect of Curcumin. As shown in **Supplementary Figure S3A**, the lincROR expression was reduced by lincROR shRNA (sh-lincROR) at the mRNA level. The SMMC-7721 cells were transfected with sh-lincROR, and we then examined cell growth. We found that lincROR knockdown led to a weaker inhibitory effect on cell growth (**Supplementary Figure S3B**) than that induced by Curcumin. On the other hand, the lincROR-overexpressing SMMC-7721 cell line was generated, and lincROR was obviously up-regulated in these stable cells (**Figure 6A**). The examination of cell viability and colony formation showed that lincROR overexpression partially reversed the Curcumin-induced cell proliferative inhibition (**Figures 6B, C**). As for the Wnt/β-catenin signaling, our results demonstrated that lincROR overexpression significantly abolished the suppressive expression of total β-catenin (**Figure 6D**, **Supplementary Figure S4**) and several downstream target genes of Wnt/β-catenin signaling (**Figure 6E**), thus partially cancelling the inactivation of Wnt/β-catenin signaling in Curcumin-treated HCC cells.

## Discussion

Traditional Chinese herbs have been used for thousands of years and continue to be popular today. Many herbs have been demonstrated to alleviate tumorigenesis, relieve the side effects of chemotherapy, or extend the patients' life (Hu et al., 2013). Curcumin is a polyphenol found in *Curcuma longa* and has been used as a spice in India for centuries (Tsuda, 2018). It has been reported to exhibit health benefits such as being antioxidant, anti-inflammatory, and having anticancer properties (Joe et al., 2004; Strimpakos and Sharma, 2008; Basnet and Skalko-Basnet, 2011). As for the anti-cancer activity, Curcumin has been demonstrated to suppress various cancers such as gastric cancer (Jakubek et al., 2019), breast cancer (Choudhuri et al., 2002; Liu and Chen, 2013), lung cancer (Hatcher et al., 2008; Chen et al., 2019), etc. In the present study, we found that Curcumin inhibited the *in vitro* HCC tumor growth through inducing cell cycle arrest and apoptosis. However, although some fragmented studies have touched on the underlying mechanism, there is a lack of systemic investigation. Therefore, depicting the detailed mechanism involved could provide a strong basis for its clinical practice in the near future.

In recent years, the emergence of noncoding RNAs (ncRNAs) bring new light for the investigation of TCM's mechanisms. In addition to miRNAs, long or large noncoding RNAs (lncRNAs) with a length of over 200 bases have recently been identified as novel regulators of gene activity (Ponting et al., 2009). A number of recent papers have revealed that lncRNAs are important and powerful regulators in various biological activities (Khalil et al., 2009). To date, several lncRNAs have been reported to mediate carcinogenesis. For example, the H19 gene induced the epithelial to mesenchymal transition (EMT) in colorectal cancer (Liang et al., 2015); HOTAIR expression is closely associated with breast cancer metastasis and the high expression of HOTAIR is a powerful predictor of eventual metastasis and death (Gupta et al., 2010). In the present study, several previously reported on lncRNAs related to tumorigenesis, including HULC, lincROR, HOTTIP, and H19, were chosen for examination of their expression profiling. Of these, lincROR was the most promising candidate in the Curcumin-treated HCC cells. The human lincROR is a recently identified 2.6-kb lincRNA located in chromosome 18 (hg19 chr18:54,721,802-54,739,350), consisting of four exons. lincROR is highly expressed in ESC and iPSCs (Loewer et al., 2010) and maintains their self-renewal (Wang et al., 2013). Interestingly, a recent study also documented that lincROR acted as a strong negative regulator of p53 and inhibited p53-mediated cell cycle arrest and apoptosis (Fan et al., 2018), indicating its likely function in tumorigenesis.

As is well known, the canonical Wnt/β-catenin pathway plays a significant role in many cellular functions including organ formation, stem cell renewal, and cell survival (Anastas and Moon, 2013; Song et al., 2018). Aberrant Wnt/β-catenin pathway activation could promote tumorigenesis and about 35% of HCC patients exhibited this signaling activation (Prasad et al., 2009; Suarez et al., 2015). Therefore, the inactivation of this Wnt/β-catenin signaling may provide an attractive therapeutic strategy for HCC patients. In our study, Curcumin was found to significantly suppress the luciferase activity of Wnt/β-catenin TOPflash in HCC cells, which was consistent with the previous report (Prasad et al., 2009). The β-catenin translocation from cytoplasm to nucleus is critical for the activation of the Wnt/β-catenin signaling, which interacts with LEF/TCF to stimulate the transcription of downstream genes such as c-Myc, CCND1, and CD44 (MacDonald et al., 2009). Our results demonstrated that the expression of nuclear β-catenin was decreased by Curcumin, and its downstream target genes were inhibited as well. Therefore, our data indicated that Curcumin exhibited anti-proliferative effects in HCC cells *via* inactivating the canonical Wnt pathway.

Several lncRNAs have been demonstrated to exert their function through activating/inactivating Wnt/β-catenin signaling. For example, lncRNA H19 could activate the Wnt/β-catenin pathway to mediate the epithelial to mesenchymal transition (EMT) and migration in colorectal cancer (Liang et al., 2015). lncRNA-NEF could antagonize cancer metastasis through inactivating Wnt/β-catenin signaling in HCC (Liang et al., 2018). Our previous study also demonstrated that lincROR could induce Wnt/β-catenin signaling and promote osteogenic differentiation of mesenchymal stem cells. In this study, lincROR overexpression partially reversed the Curcumin-induced cell proliferative inhibition, and partially abolished the inactivation of Wnt/β-catenin signaling in Curcumin-treated HCC cells. Therefore, our results demonstrated that Curcumin suppressed cell viability through linc-ROR/β-catenin regulatory pattern. Serving as a natural miRNA sponge or RNA decoy, linc-ROR was found to interact with miR-138 and miR-145 and promoted osteogenesis (Feng et al., 2018). This lncRNA could also act as a “sponge” against miR-145 to mediate the self-renewal ability of stem cells (Cheng and Lin, 2013), and mediate the differentiation of endometrial cancer stem cells (Zhou et al., 2014). More interestingly, Curcumin was reported to suppress the proliferation and tumorigenicity of prostate cancer through a ceRNA effect of miR-145 and lncRNA-ROR (Liu et al., 2017).

In summary, our results demonstrated that Curcumin plays an anti-tumor function through the linc-ROR/β-catenin regulatory pattern in HCC. This study elucidates a new mechanism of Curcumin in HCC and sheds light on developing a novel therapeutic for HCC patients, especially for patients with high levels of Wnt/β-catenin signaling. But more interestingly, although Curcumin is considered as a nutraceutical in foods and supplement products, intake of Curcumin from daily foods could be not sufficient for the anti-tumor activity because of its poor solubility and chemical instability, which results in low bioavailability. Therefore, we will try to develop a strategy to improve curcumin's bioavailability in the next study, which may facilitate the extension of its usage in clinical practice in the future.

## Data Availability Statement

The datasets generated for this study are available on request to the corresponding authors.

## Author Contributions 

J-FZ and W-MF spearheaded and supervised all the experiments. J-FZ and W-MF designed the experiments. YL, C-JS, JS, and F-WZ conducted the experiments. F-FP provided technical support. W-MF, YL, C-JS, JS, and J-FZ analyzed the data and prepared the manuscript.

## Conflict of Interest

The authors declare that the research was conducted in the absence of any commercial or financial relationships that could be construed as a potential conflict of interest.

## References

[B1] Anastas J. N. Moon R. T. (2013). WNT signalling pathways as therapeutic targets in cancer. Nat. Rev. Cancer 13, 11–26. 10.1038/nrc3419 23258168

[B2] Basnet P. Skalko-Basnet N. (2011). Curcumin: an anti-inflammatory molecule from a curry spice on the path to cancer treatment. Molecules 16 (6), 4567–4598. 10.3390/molecules16064567 21642934 PMC6264403

[B3] Bhan A. Soleimani M. Mandal S. S. (2017). Long Noncoding RNA and Cancer: A New Paradigm. Cancer Res. 77, 3965–3981. 10.1158/0008-5472.CAN-16-2634 28701486 PMC8330958

[B4] Bosch F. X. Ribes J. D´AZ M. Cléries R. (2004). Primary liver cancer: Worldwide incidence and trends. Gastroenterology 127, S5–16. 10.1053/j.gastro.2004.09.011 15508102

[B5] Chen P. Huang H. P. Wang Y. Jin J. Long W. G. Chen K. (2019). Curcumin overcome primary gefitinib resistance in non-small-cell lung cancer cells through inducing autophagy-related cell death. J. Exp. Clin. Cancer Res. 38, 254. 10.1186/s13046-019-1234-8 31196210 PMC6567416

[B6] Cheng E. C. Lin H. (2013). Repressing the repressor: a lincRNA as a MicroRNA sponge in embryonic stem cell self-renewal. Dev. Cell 25 (1), 1–2. 10.1016/j.devcel.2013.03.020 23597480 PMC3906851

[B7] Choudhuri T. Pal S. Agwarwal M. L. Das T. Sa G. (2002). Curcumin induces apoptosis in human breast cancer cells through p53-dependent Bax induction. FEBS Lett. 512, 334–340. 10.1016/S0014-5793(02)02292-5 11852106

[B8] Fan Y. Yan T. Chai Y. Jiang Y. Zhu X. (2018). Long noncoding RNA HOTTIP as an independent prognostic marker in cancer. Clin. Chim. Acta 482, 224–230. 10.1016/j.cca.2017.07.031 28778381

[B9] Feng L. Shi L. Lu Y. F. Wang B. Tang T. Fu W. M. (2018). Linc-ROR promotes osteogenic differentiation of mesenchymal stem cell through functioning as a competing endogenous RNA for miR-138 and miR-145. Mol. Ther. Nucleic Acids 11, 345–353. 10.1016/j.omtn.2018.03.004 29858070 PMC5992460

[B10] Fu W. M. Zhu X. Wang W. M. Lu Y. F. Wang H. Liang W. C. (2015). Hotair Mediates hepatocarcigenesis through suppressing miRNA-218 expression and Activating P14 and P16 Signaling. J. Hepatol. 63 (4), 886–895. 10.1016/j.jhep.2015.05.016 26024833

[B11] Golonko A. Lewandowska H. Świsłocka R. Jasińska U. T. Priebe W. Lewandowski W. (2019). Curcumin as tyrosine kinase inhibitor in cancer treatment. Eur. J. Med. Chem. 181. 10.1016/j.ejmech.2019.07.015 31404861

[B12] Gupta R. A. Shah N. Wang K. C. Kim J. Horlings H. M. Wong D. J. (2010). Long non-coding RNA HOTAIR reprograms chromatin state to promote cancer metastasis. Nature 464, 1071–1076. 10.1038/nature08975 20393566 PMC3049919

[B13] Hatcher H. Planalp R. Cho J. Torti F. M. Torti S. V. (2008). Curcumin: from ancient medicine to current clinical trials. Cell Mol. Life Sci. 65, 1631–1652. 10.1007/s00018-008-7452-4 18324353 PMC4686230

[B14] Hu Y. Wang S. Wu X. Zhang J. Chen R. Chen M. (2013). Chinese herbal medicine-derived compounds for cancer therapy: a focus on hepatocellular carcinoma. J. Ethnopharmacol. 149, 601–612. 10.1016/j.jep.2013.07.030 23916858

[B15] Jakubek M. Kejík Z. Kaplánek R. Hromádka R. Šandriková V. Sýkora D. (2019). Strategy for improved therapeutic efficiency of curcumin in the treatment of gastric cancer. BioMed. Pharmacother. 118, 109278. 10.1016/j.biopha.2019.109278 31387004

[B16] Jemal A. Ward E. Hao Y. Thun M. (2005). Trends in the leading causes of death in the United States 1970-2002. JAMA 294, 1255–1259. 10.1001/jama.294.10.1255 16160134

[B17] Joe B. Vijaykumar M. Lokesh B. R. (2004). Biological properties of curcumin-cellular and molecular mechanisms of action. Crit. Rev. Food Sci. Nutr. 44, 97–111. 10.1080/10408690490424702 15116757

[B18] Khalil A. M. Guttman M. Huarte M. Garber M. Raj A. Rivea Morales D. (2009). Many human large intergenic noncoding RNAs associate with chromatin-modifying complexes and affect gene expression. Proc. Natl. Acad. Sci. U.S.A. 106, 11667–11672. 10.1073/pnas.0904715106 19571010 PMC2704857

[B19] Lecerf C. Le Bourhis X. Adriaenssens E. (2019). The long non-coding RNA H19: an active player with multiple facets to sustain the hallmarks of cancer. Cell Mol. Life Sci. 76 (23), 4673–4687. 10.1007/s00018-019-03240-z 31338555 PMC11105575

[B20] Liang W. C. Fu W. M. Wang Y. Wang W. M. Wang Y. B. Jiang H. Q. (2015). The LncRNA H19 Promotes Epithelial to Mesenchymal Transition by Functioning as ceRNA in Colorectal Cancer. Oncotarget 6, 22513–22525. 10.18632/oncotarget.4154 26068968 PMC4673179

[B21] Liang W. C. Ren J. L. Wong C. K. Chen S. O. Waye M. M. Fu W. M. (2018). The LncRNA-NEF antagonized epithelial to mesenchymal transition and cancer metastasis via cis-regulating FOXA2 and inactivating Wnt/β-catenin signaling. Oncogene 37, 1445–1456. 10.1038/s41388-017-0041-y 29311643

[B22] Liang W. C. Liang P. P. Shi M. Cao Y. Rao S. T. Tsui S. K. (2019). Translation of circular RNA circβ-catenin promotes liver cancer cell growth through activation of Wnt/β-catenin pathway. Genome Biol. 20, 84. 10.1186/s13059-019-1685-4 31027518 PMC6486691

[B23] Liu D. Chen Z. (2013). The effect of curcumin on breast cancer cells. J. Breast Cancer 16 (2), 133–137. 10.4048/jbc.2013.16.2.133 23843843 PMC3706856

[B24] Liu T. Chi H. Chen J. Chen C. Huang Y. Xi H. (2017). Curcumin suppresses proliferation and in vitro invasion of human prostate cancer stem cells by ceRNA effect of miR-145 and lncRNA-ROR. Gene 631, 29–38. 10.1016/j.gene.2017.08.008 28843521

[B25] Loewer S. Cabili M. N. Guttman M. Loh Y. H. Thomas K. Park I. H. (2010). Large intergenic non-coding RNA-RoR modulates reprogramming of human induced pluripotent stem cells. Nat. Genet. 42, 1113–1117. 10.1038/ng.710 21057500 PMC3040650

[B26] MacDonald B. T. Tamai K. He X. (2009). Wnt/beta-catenin signaling: components, mechanisms, and diseases. Dev. Cell 17, 9–26. 10.1016/j.devcel.2009.06.016 19619488 PMC2861485

[B27] Mercer T. R. Dinger M. E. Mattick J. S. (2009). Long non-coding RNAs: insights into functions. Nat. Rev. Genet. 10, 155–159. 10.1038/nrg2521 19188922

[B28] Okuda K. (2000). Hepatocellular carcinoma. J. Hepatol. 32, 225–237. 10.1016/S0168-8278(00)80428-6 10728807

[B29] Ponting C. P. Oliver P. L. Reik W. (2009). Evolution and functions of long noncoding RNAs. Cell 136 (4), 629–641. 10.1016/j.cell.2009.02.006 19239885

[B30] Prasad C. P. Rath G. Mathur S. Bhatnagar D. Ralhan R. (2009). Potent growth suppressive activity of curcumin in human breast cancer cells: Modulation of Wnt/beta-catenin signaling. Chem. Biol. Interact. 181, 263–271. 10.1016/j.cbi.2009.06.012 19573523

[B31] Song J. Xie C. Jiang L. Wu G. Zhu J. Zhang S. (2018). Transcription factor AP-4 promotes tumorigenic capability and activates the Wnt/beta-catenin pathway in hepatocellular carcinoma. Theranostics 8, 3571–3583. 10.7150/thno.25194 30026867 PMC6037031

[B32] Strimpakos A. S. Sharma R. A. (2008). Curcumin: preventive and therapeutic properties in laboratory studies and clinical trials. Antioxid. Redox Signal 10, 511–545. 10.1089/ars.2007.1769 18370854

[B33] Suarez M. I. Uribe D. Jaramillo C. M. Osorio G. Perez J. C. Lopez R. (2015). Wnt/beta-catenin signaling pathway in hepatocellular carcinomas cases from Colombia. Ann. Hepatol. 14, 64–74. 10.1016/S1665-2681(19)30802-6 25536643

[B34] Tsuda T. (2018). Curcumin as a functional food-derived factor: degradation products, metabolites, bioactivity, and future perspectives. Food Funct. 9, 705–714. 10.1039/C7FO01242J 29206254

[B35] Vallée A. Lecarpentier Y. Vallée J. N. (2019). Curcumin: a therapeutic strategy in cancers by inhibiting the canonical WNT/β-catenin pathway. J. Exp. Clin. Cancer Res. 38, 323. 10.1186/s13046-019-1320-y 31331376 PMC6647277

[B36] Wang Y. Xu Z. Jiang J. Xu C. Kang J. Xiao L. (2013). Endogenous miRNA sponge lincRNA-RoR regulates Oct4, Nanog, and Sox2 in human embryonic stem cell self-renewal. Dev. Cell 25, 69–80. 10.1016/j.devcel.2013.03.002 23541921

[B37] Zhang A. Zhou N. Huang J. Liu Q. Fukuda K. Ma D. (2013). The human long non-coding RNA-RoR is a p53 repressor in response to DNA damage. Cell Res. 23, 340–350. 10.1038/cr.2012.164 23208419 PMC3587705

[B38] Zhou X. Gao Q. Wang J. Zhang X. Liu K. Duan Z. (2014). Linc-RNA-RoR acts as a “sponge” against mediation of the differentiation of endometrial cancer stem cells by microRNA-145. Gynecol. Oncol. 133 (2), 333–339. 10.1016/j.ygyno.2014.02.033 24589415

