# Supplementary figures and images for: LincROR Mediates the Suppressive Effects of Curcumin on Hepatocellular Carcinoma Through Inactivating Wnt/β-Catenin Signaling
Source: Front Pharmacol. 2020 Jul 2;11:847. doi: 10.3389/fphar.2020.00847 (PMC7351502; doi:10.3389/fphar.2020.00847)

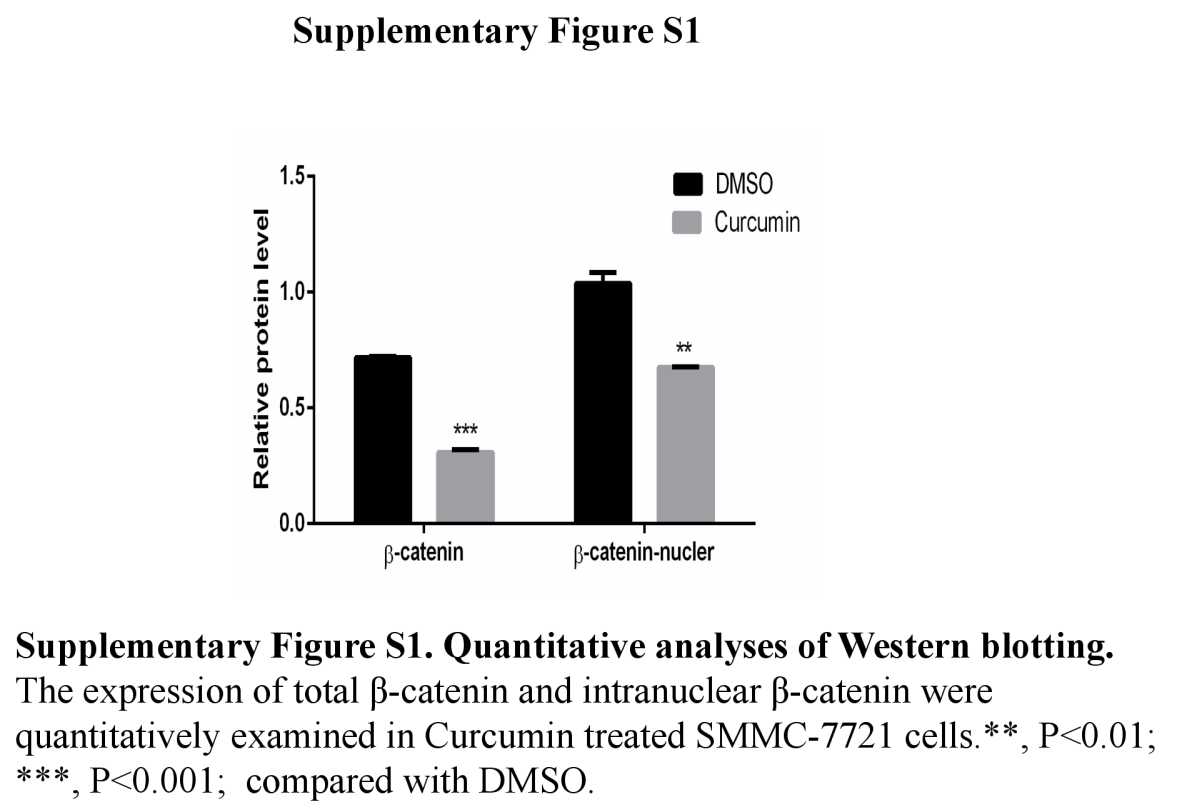

Supplement: Supplementary file 1 [file Image_1.jpeg]

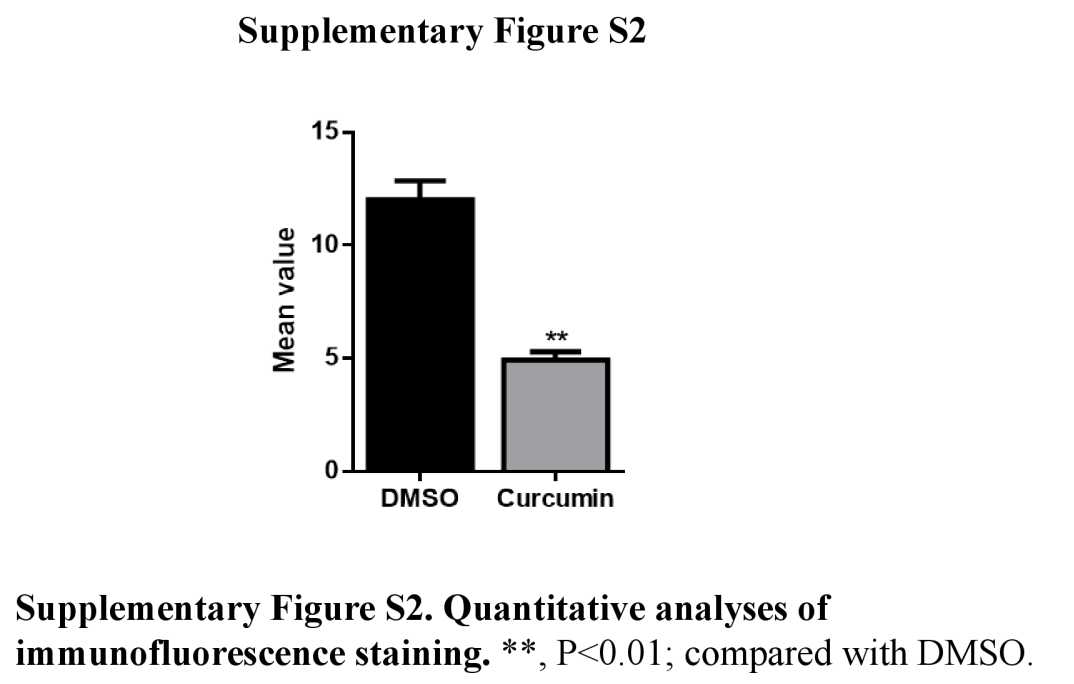

Supplement: Supplementary file 2 [file Image_2.jpeg]

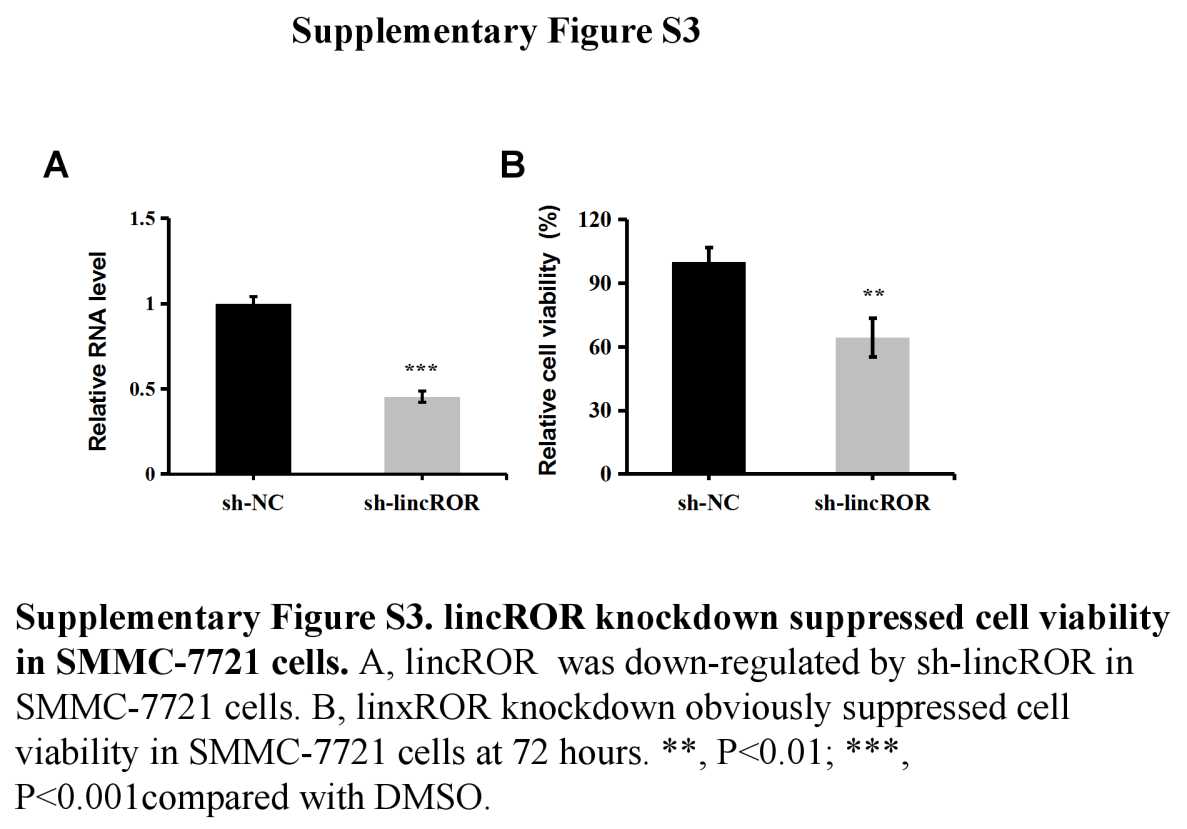

Supplement: Supplementary file 3 [file Image_3.jpeg]

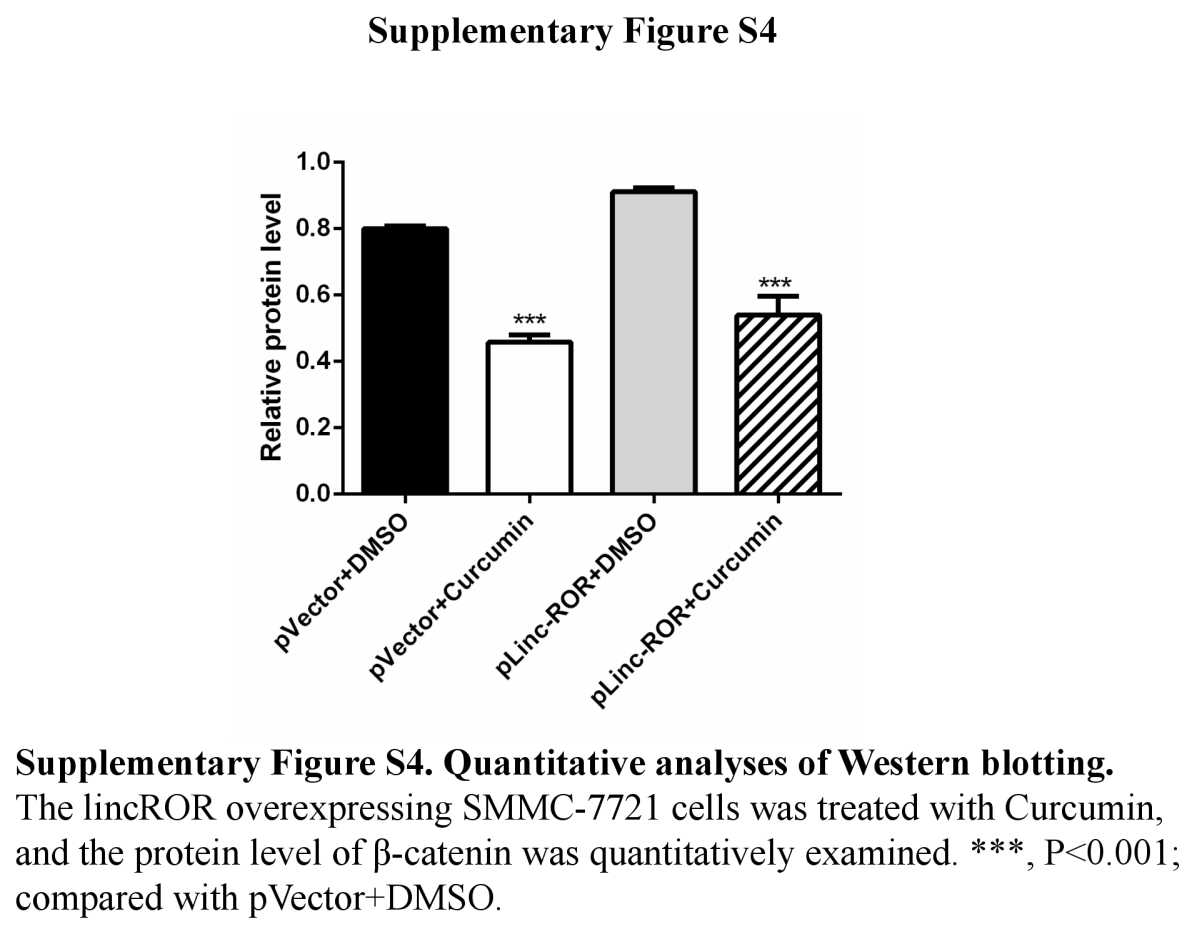

Supplement: Supplementary file 4 [file Image_4.jpeg]
